# Characterization of *Streptococcus mitis* subsp. *carlssonii* isolated from human vagina: prevalence, phenotypic, and genomic insights

**DOI:** 10.3389/fmicb.2025.1625724

**Published:** 2025-08-25

**Authors:** Jake Adolf V. Montecillo, Jung Hwa Lee, Heon Jong Yoo, Yoo-Young Lee, Chul Min Park, Angela Cho, Hyunsu Lee, Jong Mi Kim, Nan Young Lee, Sun-Hyun Park, Nora Jee-Young Park, Hyung Soo Han, Gun Oh Chong, Incheol Seo

**Affiliations:** ^1^Department of Immunology, School of Medicine, Kyungpook National University, Daegu, Republic of Korea; ^2^Clinical Omics Institute, Kyungpook National University, Daegu, Republic of Korea; ^3^Brain Korea 21 FOUR Program, School of Medicine, Kyungpook National University, Daegu, Republic of Korea; ^4^Chungnam National University School of Medicine, Daejeon, Republic of Korea; ^5^Gynecologic Cancer Center, Department of Obstetrics and Gynecology, Samsung Medical Center, Sungkyunkwan University School of Medicine, Seoul, Republic of Korea; ^6^Department of Obstetrics and Gynecology, Jeju National University Hospital, Jeju National University College of Medicine, Jeju, Republic of Korea; ^7^Department of Physiology, Pusan National University School of Medicine, Yangsan, Republic of Korea; ^8^Department of Obstetrics and Gynecology, Kyungpook National University Chilgok Hospital, Daegu, Republic of Korea; ^9^Department of Clinical Pathology, School of Medicine, Kyungpook National University, Daegu, Republic of Korea; ^10^Division of Advanced Predictive Research, Center for Bio-Signal Research, Korea Institute of Toxicology (KIT), Daejeon, Republic of Korea; ^11^Department of Pathology, Kyungpook National University Chilgok Hospital, Daegu, Republic of Korea; ^12^Department of Physiology, School of Medicine, Kyungpook National University, Daegu, Republic of Korea

**Keywords:** *Streptococcus*, microbiome, vaginal microbiome, genomics, phylogenomics

## Abstract

The genus *Streptococcus* is a heterogenous group of commensal and pathogenic bacteria. Members of this genus are classified into two major groups, the pyogenic group and the viridans group streptococci (VGS). VGS are frequently found as normal members of the human microbiome and are regarded as commensals. In this work, we characterized a VGS strain isolated from the vaginal swab sample of a Korean patient diagnosed with endometrial cancer. Phylogenetic, phenotypic, and genome-based analyses confirmed the classification of the strain K0074 as a member of the *S. mitis* complex. Based on the phylogenetic analyses, the species belonged to the recently established *Streptococcus mitis* subsp. *carlssonii*. The strain was found to be rare in the vaginal microbiome, but prevalent in the oral and nasal microbiome samples. K0074 neither triggered an inflammatory response nor caused cytolytic and cytotoxic effects on human cervical cancer cell line. Genome analysis uncovered the genetic basis of the strain's metabolism, virulence factors, and potential antimicrobial resistance phenotypes. Moreover, comparative genomics of the strain and closely related species and subspecies highlighted their striking differences in gene properties and biological functions. Overall, the new strain exhibited low virulence and pathogenic potentials and thus, is regarded as a commensal member of the human microflora. The genetic divergence of K0074 from closely related strains offers a compelling foundation for future investigations into the strain's potential functional specialization and its adaptation within the vaginal microbiome.

## 1 Introduction

The genus *Streptococcus* is a heterogenous group of Gram-positive bacteria within the phylum *Bacillota*, class *Bacilli*, order *Lactobacillales*, and family *Streptococcaceae* (Meier-Kolthoff et al., 2022). Members of the genus exhibit cocci or spherical cells that are typically arranged in pairs or chains, and are catalase-negative, non-motile, facultatively anaerobic, non-sporulating, homofermentative, and require complex nutrients for growth (Du Toit et al., 2014). The genus is considered part of the lactic acid bacteria (LAB), a group of Gram-positive bacteria that produce lactic acid as the major end product of carbohydrate fermentation (Du Toit et al., 2014; Wang et al., 2021). As of December 2024, the genus *Streptococcus*, with its type species *Streptococcus pyogenes*, is composed of 126 species with validly published and correct names as reflected in the List of Prokaryotic names with Standing in Nomenclature (LPSN) website (Meier-Kolthoff et al., 2022). These species exhibit diversity in their phylogeny, ecology, and metabolism, and can function as either commensals or pathogens (Du Toit et al., 2014; Patel and Gupta, 2018; Stoneham et al., 2021; Nanayakkara et al., 2023).

Streptococci are conventionally classified based on their hemolytic reactions on blood agar, which divided the members of the genus into two groups: the beta-hemolytic pyogenic group and the alpha-hemolytic viridans group. Beta-hemolytic species are characterized by their ability to completely lyse erythrocytes, while alpha-hemolytic are those species that displayed incomplete hemolysis (Sinner and Tunkel, 2015; Patel and Gupta, 2018). Subsequent phylogenetic analysis based on 16S rRNA gene sequences further divided the members of the genus into six groups. These groups comprised the previously identified pyogenic group, and five viridans subgroups – mitis, mutans, anginosus, salivarius, and bovis (Du Toit et al., 2014). These groups of streptococci are characterized by their distinct phenotypes and pathogenic potentials. The pyogenic group encompasses most species that are pathogenic including the highly pathogenic *S. pyogenes* and *Streptococcus agalactiae*, which are commonly known as group A *Streptococcus* (GAS) and group B *Streptococcus* (GBS), respectively (Gergova et al., 2024). On the other hand, the viridans group streptococci (VGS) represent the largest part of known streptococci and are frequently found as normal members of the human oral, gastrointestinal, and urogenital microbiome. With the exception of *Streptococcus pneumoniae*, most viridans streptococci are commensals, and are characterized to have low pathogenic potentials (Doern and Burnham, 2010; Du Toit et al., 2014; Stoneham et al., 2021; Nanayakkara et al., 2023).

In the human vagina, streptococci are represented by GBS and several members of the VGS (Török and Day, 2014; Brokaw et al., 2021; Stoneham et al., 2021; Gergova et al., 2024). GBS colonization in the vagina, especially during pregnancy, is associated with adverse gynecological and obstetric infections (Brokaw et al., 2021; Gergova et al., 2024). GBS possess an arsenal of virulence factors such as capsules, adhesins, enzymes, and exotoxins that aid in their successful colonization, persistence, and evasion of host immune responses (Gergova et al., 2024). Unlike GBS, VGS colonization in the vagina has not been implicated with adverse gynecological outcomes and in general, are rarely associated with severe infections (Denapaite et al., 2016).

In this work, we characterized a new strain (K0074) of *Streptococcus mitis* subsp. *carlssonii*, a member of the VGS, isolated from the vaginal swab sample of a Korean patient diagnosed with endometrial cancer and with no signs of gynecological bacterial infections. The strain was found to be rare in the vaginal microbiome samples and did not elicit an inflammatory response or induce cytolytic and cytotoxic effects on human cervical cancer cell line. Comparative genomic analysis of strain K0074 uncovered the genetic basis of its metabolism, virulence factors, and potential antimicrobial resistance phenotypes. Based on the demonstrated phenotypic and genetic features, *S. mitis* subsp. *carlssonii* K0074 is considered to have low pathogenic potential, particularly when compared to its closely related pathogenic species, *S. pneumoniae*.

## 2 Material and methods

### 2.1 Sample collection and bacterial isolation

The vaginal swab sample used in this study was part of the collection of vaginal swab samples taken from Korean women participating in the “Hospital-Based Human Microbiome R&D Project”. The study protocol of the project was reviewed and approved by the Institutional Review Committee of Kyungpook National University Chilgok Hospital (KNUCH 2023-08-054-011). All individuals enrolled in the project provided signed informed consent, authorizing the collection and use of their medical information and biological samples for research purposes. Procurement of the vaginal swab samples was performed as previously described (Montecillo et al., 2024). Isolation of the bacteria from the swab sample was carried out on de Man, Rogosa, and Sharpe (MRS) agar supplemented with 4 mM of L-cysteine at 37 °C under aerobic condition for 24–74 h as detailed in the previous study (Montecillo et al., 2024).

### 2.2 16S rRNA gene sequencing

Pure cultures of the K0074 isolate on agar plates were sent to Macrogen, South Korea, for DNA extraction and sequencing of the 16S rRNA gene using the universal primers 27F and 1492R. The resulting nearly complete 16S rRNA gene sequence was then subjected to a similarity-based search against the quality-controlled 16S rRNA gene sequence database of EzBioCloud (Yoon et al., 2017a).

### 2.3 Phylogenetic analyses

Phylogenetic trees were reconstructed based on two different sequence datasets, the 16S rRNA gene and the whole-genome sequences (WGSs). The phylogenetic tree based on the 16S rRNA gene sequences was reconstructed using MEGA 11 (Tamura et al., 2021). The sequence alignment file obtained from the EzBioCloud's identification service containing the 16S rRNA gene sequences of the most closely related taxa of K0074 was utilized for the analysis. To infer the evolutionary relationships and genetic distances, the maximum likelihood (ML) method (Thorne et al., 1991) and Kimura's two-parameter model (Kimura, 1980) were used, respectively. The branch support values were based on 1000 bootstrap replicates. The genome-based phylogenetic tree was reconstructed using the Type (Strain) Genome Server (TYGS) employing the genome BLAST distance phylogeny (GBDP) approach (Meier-Kolthoff and Göker, 2019). The resulting phylogenetic trees were then visualized using the Interactive tree Of Life (iTOL) (Letunic and Bork, 2024).

### 2.4 Genome sequencing and annotation

Whole-genome sequencing of K0074 was performed at Sanigen Co., Ltd, Republic of Korea using their hybrid sequencing platform composed of MiSeq (Illumina) and Nanopore (Oxford Nanopore Technologies) sequencing technologies. The sequence data were processed and assembled *de novo* using Unicycler v0.4.8 and the PathoSystems Resource Integration Center (PATRIC) v3.6.12 online pipeline (Davis et al., 2020). Gene annotations were performed using the NCBI Prokaryotic Genome Annotation Pipeline (PGAP) (Tatusova et al., 2016). Functional annotation and clusters of orthologous gene (COG) assignments were conducted using eggNOG-mapper v2 (Cantalapiedra et al., 2021). Metabolic pathways were identified by mapping the protein sequences against the Kyoto Encyclopedia of Genes and Genomes (KEGG) database using BlastKOALA (Kanehisa et al., 2016). The graphical representation of the WGS was generated using Proksee (Grant et al., 2023).

### 2.5 Physiological and biochemical characterization

The phenotypic characterization of K0074, unless otherwise stated, was performed using cells grown on Difco Brain Heart Infusion (BHI) agar or broth at 37 °C under aerobic conditions for 24–74 h. Gram-staining was performed using Gram-staining kit (MBcell). Growth at various pH, NaCl (w/v), and temperature was determined using BHI broth medium. A range of pH values (pH 3.0–8.0) was achieved by adjusting the pH of the medium using HCl or 1N NaOH. Catalase activity was assessed using 3.0% (v/v) hydrogen peroxide. Production of gelatinase was examined using BHI agar supplemented with gelatin (120 g/L). The production of other enzymes and the utilization of sugars were assessed using VITEK 2 System (bioMérieux). For comparative analyses with closely related strains, the type strain of *Streptococcus mitis* subsp. *mitis* KCTC 13047^T^ and strains of *Streptococcus mitis* subsp. *carlssonii* (formerly *Streptococcus hohhotensis* KCTC 21155^T^ and *Streptococcus humanilactis* KCTC 21157^T^), were purchased from Korean Collection for Type Cultures (KCTC) and included in the phenotypic characterization.

### 2.6 Cytolytic and cytotoxic characterization

The human cervical carcinoma HeLa cells (KCLB 10002) were obtained from the Korean Cell Line Bank (KCLB). The cells were maintained in Dulbecco's Modified Eagle Medium (DMEM) with 10% fetal bovine serum (FBS) and 1% penicillin-streptomycin at 37 °C and 5% CO_2_. Resazurin assay (Petiti et al., 2024) was performed to determine the effect of the cell-free supernatant (CFS) of K0074 on HeLa cell's proliferation. To assess the cytolytic effect of the CFS, trypan blue exclusion assay (Strober, 2015) was employed. The effect of the CFS on the migration ability of HeLa cells was examined using scratch assay as previously described (Liang et al., 2007). The CFS of K0074 was prepared from the 24-h grown culture in MRS medium. The culture supernatant was collected following centrifugation at 10,000 rpm for 3 min, which was then filtered by passing through a 0.2 μm membrane filter (Sartorius). The resulting CFS was immediately used for the assays or stored at −20 °C. The assays were performed in a 96-well plate for 24 h with 1 × 10^4^ HeLa cells and 25% CFS.

Expression of pro-inflammatory genes was quantified through quantitative reverse transcription-PCR (qRT-PCR). Total RNA from HeLa cells was extracted using TRIzol reagent (Invitrogen) following the manufacturer's instructions. The purified RNA samples were treated with DNase following the instructions provided in the Turbo DNA-free kit (Invitrogen). For cDNA synthesis, the High-Capacity cDNA Reverse Transcription Kit (Applied Biosystems) was used. Briefly, 1 μg of RNA was combined with a mixture containing buffer, dNTPs, random primers, reverse transcriptase enzyme, and RNase inhibitor. RNase-free water was then added to adjust the final volume to 20 μL. The mixture was exposed to a series of incubation steps: 25 °C for 10 min, 37 °C for 120 min, and 85 °C for 5 min. The synthesized cDNA was subsequently stored at −20 °C until further use. For qPCR analysis, 1 μL of the cDNA was mixed with TOPreal SYBR Green qPCR High-ROX Premix (Enzynomics) and analyzed using the QuantStudio 3 Real-Time PCR System (Applied Biosystems). The primers for the target genes are listed in the Supplementary Table 1. The expressions of the target genes relative to the expression of the internal control gene were determined using the 2^−ΔCT^ method (Schmittgen and Livak, 2008).

### 2.7 Detection of K0074 across human microbiome samples

The prevalence of K0074 in the human microbiome was predicted based on the taxonomic assignments of the 16S rRNA gene sequences data encompassing various human body sites. The publicly available 16S rRNA gene sequences datasets were downloaded from the NCBI Sequence Read Archive (SRA) database in December 2024. The accession numbers are listed in the Supplementary Table 2. Quality filtering and taxonomic assignments of the 16S rRNA gene sequences datasets were performed using MOTHUR v1.48.2 software (Schloss et al., 2009) with the EzBioCloud 16S rRNA gene sequences database (Chalita et al., 2024). The detailed procedure for the analysis can be found at https://help.ezbiocloud.net/how-to-use-ezbiocloud-16s-database-with-mothur-2/.

### 2.8 Comparative genomics

Genome sequences of the closely related *Streptococcus* strains and type strains of species were retrieved from the NCBI Datasets database in November 2024. The genome accession numbers are listed in the Supplementary Table 3. The OrthoANIu (Yoon et al., 2017b) of the EzBioCloud tool was used to determine the pairwise average nucleotide identity (ANI) values. The digital DNA-DNA hybridization (dDDH) values among the genome sequences were calculated using formula *d*_4_of the Genome-to-Genome Distance Calculator (GGDC) (Meier-Kolthoff et al., 2014). To identify orthologous gene clusters (orthogroups), the OrthoFinder v3.0 tool was used (Emms and Kelly, 2019). Virulence factors and antimicrobial resistance genes (AMR) were identified using ABRicate (Seemann, n.d.) equipped with the virulence factor database (VFDB) (Jia et al., 2017) and Comprehensive Antibiotic Resistance Database (CARD) (Jia et al., 2017). Putative genes acquired through horizontal gene transfer (HGT) from the genome sequences were predicted using HGTector (Zhu et al., 2014) with the default settings. Carbohydrate active enzymes (CAZymes) were identified using dbCAN3 (Zheng et al., 2023a). The completeness of the metabolic pathways based on KEGG annotation was assessed using KEGG-Decoder (Graham et al., 2018). Hierarchical clustering was performed using SRplot (Tang et al., 2023) with Euclidean distance method. Graphical representations were generated using jvenn (Bardou et al., 2014) and ChiPlot (https://www.chiplot.online/).

## 3 Results

### 3.1 Isolation, genome analysis, and identification of strain K0074

**Figure 1:**
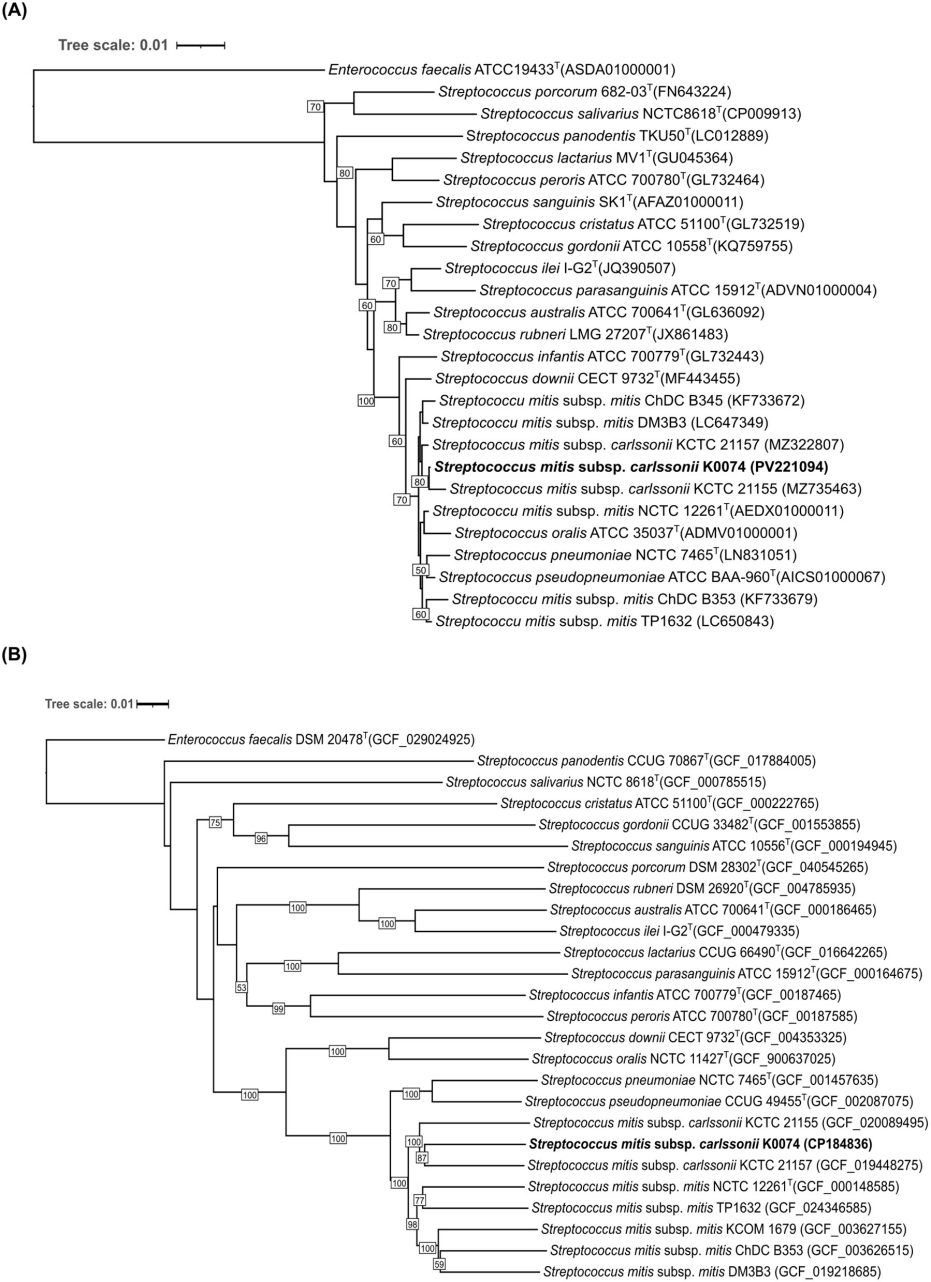
Phylogenetic tree of K0074 and closely related strains and species of *Streptococcus*. **(A)** 16S rRNA-based phylogenetic tree showing the phylogenetic position of K0074 within the genus *Streptococcus*. The tree was reconstructed using ML method with bootstrap support values based on 1,000 replicates indicated at the branching points. The tree is drawn to scale with branch lengths measured in the number of substitutions per site. *Enterococcus faecalis* ATCC 19433^T^ was used as an outgroup. **(B)** Phylogenomic tree inferred with FastME 2.1.6.1 from GBDP distances calculated from the genome sequences showing the phylogenetic position of K0074 within the genus *Streptococcus*. The branch lengths are scaled in terms of GBDP distance formula *d*_5_. The numbers at the branches are GBDP pseudo-bootstrap support values >50% from 100 replications. *Enterococcus faecalis* DSM 20478^T^ was used as an outgroup.

Strain K0074 was obtained from the human vaginal swab sample of a patient with endometrial cancer. The strain was isolated using MRS agar medium under aerobic condition. The colonies were small, round, and translucent, with a smooth, moist texture and an entire margin. Microscopic observation of the strain revealed Gram-positive coccoid cells arranged in long chains (Supplementary Figures 1, 2). Analysis of the nearly complete sequence of the 16S rRNA gene of strain K0074 demonstrated close identity to the members of the genus *Streptococcus* (Supplementary Table 4). The strain showed the highest sequence similarity to an unclassified species of *Streptococcus* (99.93%). Among the species of *Streptococcus* with validly published names, the strain exhibited highest sequence similarities to the strains of *Streptococcus mitis* subsp. *carlssonii*, formerly known as *Streptococcus hohhotensis* KCTC 21155 (99.85%) and *Streptococcus humanilactis* KCTC 21157 (99.78%). Phylogenetic analysis based on 16S rRNA gene sequences clustered the strain K0074 along with the established members of the VGS, particularly within the *S. mitis* complex (Figure 1A). The strain formed a well-supported clade with its closely related strains of *S. mitis* subsp. *cralssonii* (KCTC 21155 and KCTC 21157). Consequently, in the genome-based phylogenetic tree (Figure 1B), strain K0074 also formed a robust clade with KCTC 21155 and KCTC 21157, albeit in a different topology. In contrast to the 16S rRNA-based phylogenetic tree, the genome-based phylogenetic tree revealed that the strain K0074 was more phylogenetically closely related to strain KCTC 21157 than KCTC 21155. Strain K0074 and KCTC 21157 were observed to form a highly supported tight clade. Both phylogenetic trees demonstrated the affiliation of K0074 with the mitis subgroup of the VGS.

**Figure 2:**
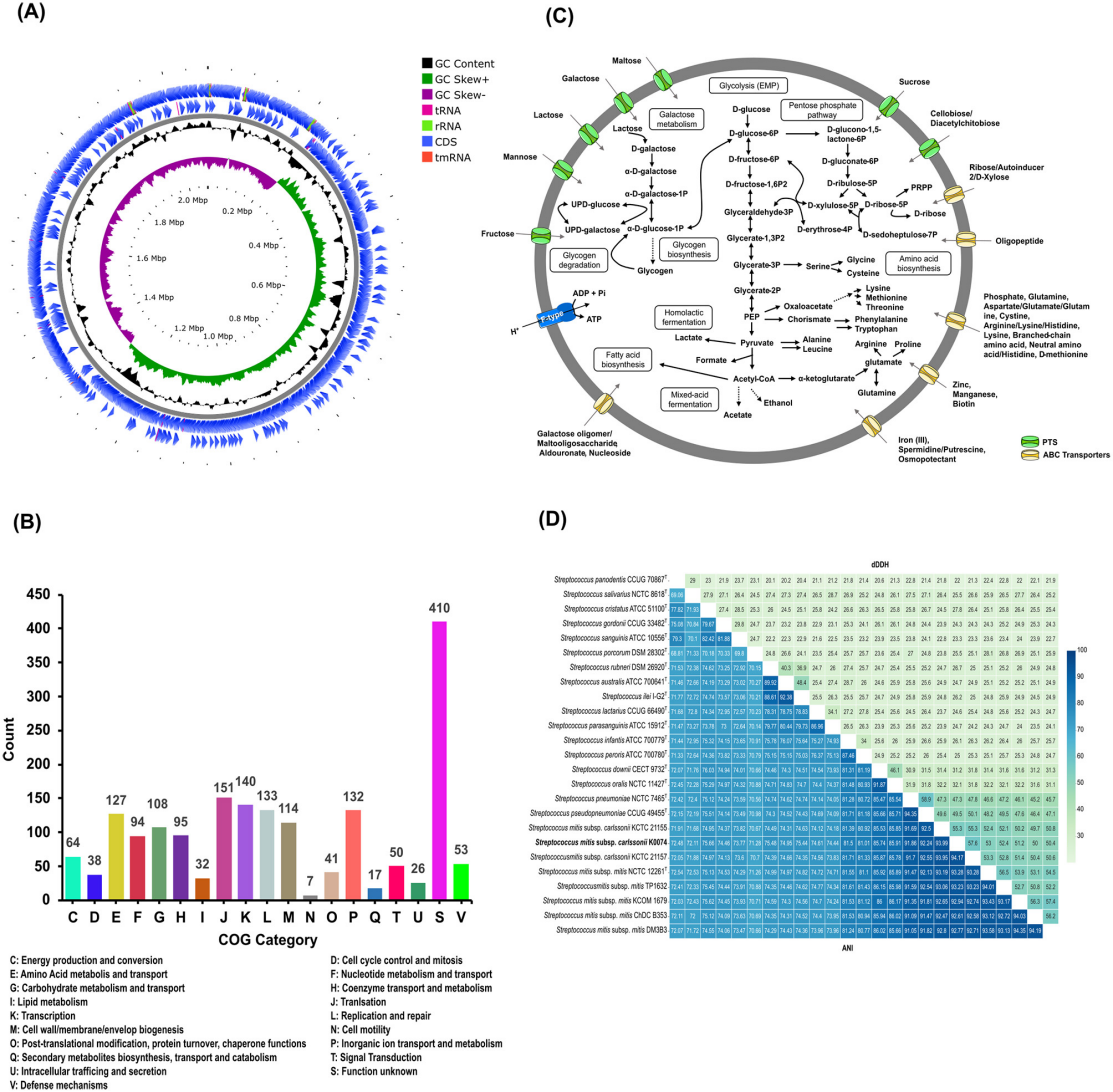
Genome features and analysis of K0074. **(A)** Circular genome map with marked characteristics constructed based on the sequenced genome of strain K0074. **(B)** COG classifications of the coding sequences from the genome of K0074. **(C)** Reconstructed metabolic pathways of K0074 inferred from the genome sequence. **(D)** Pairwise dDDH and ANI of K0074 with closely related strains and species of *Streptococcus* calculated from the genome sequences. Individual values are indicated and are also depicted as color gradient.

The whole genome of strain K0074 was sequenced with a high BUSCO completeness (100%). The whole genome sequence (WGS) of the strain is composed of a single circular chromosome with a size of 2,077,511 bp and a G+C content of 40.10%. The WGS of K0074 comprised 1,985 coding sequences (CDS), 12 rRNAs, and 60 tRNAs. Out of the identified 1,985 CDS, 1,832 were successfully assigned to specific clusters of orthologous genes (COGs), accounting for 92.25% of the total CDS. The remaining 154 CDS (7.75%) failed to correspond to any currently known COGs and hence were unclassified. Excluding the unassigned CDS, the top highly represented COGs were function unknown (22.38%), translation (8.24%), transcription (7.64%), and replication and repair (7.26%) corresponding to COG S, J, K, L, respectively. The circular map of the WGS and the proportion of the identified COGs are shown in Figures 2A, B.

The WGS of K0074 was identified to contain genes encoding an intact glycolytic Embden-Meyerhof-Parnas (EMP) pathway, pentose phosphate pathway, and genes involved in the pyruvate oxidation, galactose/lactose metabolism, glycogen biosynthesis and degradation, biosynthesis of amino acids, production of acids (lactate, formate, and acetate), and fatty acid biosynthesis. Several transporters including the sugar transporters phosphoenolpyruvate: sugar phosphotransferase system (PTS) and ATP-binding cassette (ABC) transporters, were also identified in the WGS of strain K0074. Genes responsible for the formation of a proton-translocating pump, the F-ATPase/synthase, were also identified. On the other hand, no genes encoding a complete tricarboxylic acid (TCA) cycle were identified in the WGS of the strain, a characteristic shared by all species within the genus *Streptococcus*. The predicted major metabolic pathways of the strain K0074 reconstructed based on the WGS are illustrated in Figure 2C.

Determination of the digital DNA-DNA hybridization (dDDH) values using the WGS of K0074 demonstrated values less than 70% with its known closely related species and strains (Figure 2D). The average nucleotide identity (ANI) values exhibited by K0074 with its closely related species and strains were below 95%. The shared dDDH and ANI values among all related species of *Streptococcus* ranged from 20.10–58.90% and 68.81–94.35%, respectively. Notably, K0074 exhibited 55.30–57.60% dDDH and 93.99–94.17% ANI values with the phylogenetically closely related strains of *S. mitis* subsp. *carlssonii*. These values aligned with the classification scheme for the *S. mitis* complex recently proposed by Kilian et al. (2025), supporting the identification of K0074 as a strain of *S. mitis* subsp. *carlssonii*.

Phenotypic characterization of the strain K0074 revealed distinct features in comparison to its closest phylogenetically related strains KCTC 21155 and KCTC 21157, and also distinguishable from *S. mitis* subsp. *mitis* (Table 1). Compared to the other strains, K0074 exhibited positive reactions in the utilization of D-galactose, lactose, D-mannose, N-acetyl-D-glucosamine, and salicin, as well as in the production of α-glucosidase enzyme. The other strains were also observed to have their own unique features, distinguishing them from the strain K0074. Taken together, our results revealed strong genetic and phenotypic basis for classifying K0074 as a distinct strain of *S. mitis* subsp. *carlssonii*.

**Table 1:** Phenotypic characterization of K0074 and closely related strains.

**Characteristics**	**1**	**2**	**3**	**4**
Gram stain	**+**	**+**	**+**	**+**
pH	6.0–8.0	6.0–8.0	5.0–8.0	5.0–8.0
NaCl 2% (w/v)	**+**	**+**	**+**	**+**
Temperature 25 °C	**-**	**-**	**-**	**+**
Catalase	**-**	**-**	**-**	**-**
Gelatinase	**-**	**-**	**-**	**-**
D-Amygdalin	**+**	**+**	**-**	**+**
Phosphatidylinositol phospholipase C	**-**	**-**	**-**	**-**
D-Xylose	**-**	**-**	**-**	**-**
Arginine Dihydrolase 1	**-**	**-**	**-**	**-**
β-Galactosidase	**-**	**-**	**-**	**-**
α-Glucosidase	**+**	**-**	**-**	**-**
Ala Phe Pro arylamidase	**+**	**+**	**-**	**+**
Cyclodextrin	**-**	**-**	**-**	**-**
L-Aspartate arylamidase	**-**	**-**	**+**	**-**
β-Galactopyranosidase	**-**	**-**	**+**	**-**
α-Mannosidase	**-**	**-**	**-**	**-**
Phosphatase	**-**	**+**	**-**	**-**
Leucine arylamidase	**+**	**+**	**+**	**+**
L-Proline arylamidase	**-**	**-**	**-**	**-**
β-Glucaronidase	**-**	**-**	**-**	**-**
α-Galactosidase	**-**	**-**	**+**	**-**
L-Pyrrolidonyl-arylamidase	**-**	**-**	**-**	**-**
β-Glucaronidase	**-**	**-**	**-**	**-**
Alanine arylamidase	**+**	**+**	**+**	**+**
Tyrosine arylamidase	**+**	**+**	**+**	**+**
D-Sorbitol	**-**	**-**	**-**	**-**
Urease	**-**	**-**	**-**	**-**
Polymixin B resistance	**-**	**-**	**-**	**-**
D-Galactose	**+**	**-**	**-**	**-**
D-Ribose	**-**	**-**	**-**	**-**
L-Lactate alkalinization	**-**	**-**	**-**	**-**
Lactose	**+**	**-**	**-**	**-**
N-Acetyl-D-glucosamine	**+**	**-**	**-**	**-**
D-Maltose	**+**	**-**	**-**	**+**
Bacitracin resistance	**-**	**-**	**-**	**-**
Novobiocin resistance	**-**	**-**	**-**	**-**
Growth in 6.5% NaCl	**-**	**-**	**-**	**-**
D-Mannitol	**-**	**-**	**-**	**-**
D-Mannose	**+**	**-**	**-**	**-**
Methyl-B-D-glucopyranoside	**-**	**-**	**-**	**-**
Pullulan	**-**	**-**	**-**	**-**
D-Raffinose	**+**	**-**	**-**	**+**
Salicin	**w**	**-**	**-**	**-**
Saccharose/sucrose	**+**	**-**	**-**	**+**
D-Trehalose	**+**	**+**	**-**	**-**
Arginine dihydrolase 2	**-**	**-**	**-**	**-**

Strain 1, K0074; 2, *S. mitis* subsp. *carlssonii* KCTC 21155; 3, *S. mitis* subsp. *carlssonii* KCTC 21157; 4, *S. mitis* subsp. *mitis* KCTC 13047^T^. All data were obtained from this study. +, positive; –, negative; w, weak.

### 3.2 K0074 is rare in the vagina but prevalent in other human body sites

**Figure 3:**
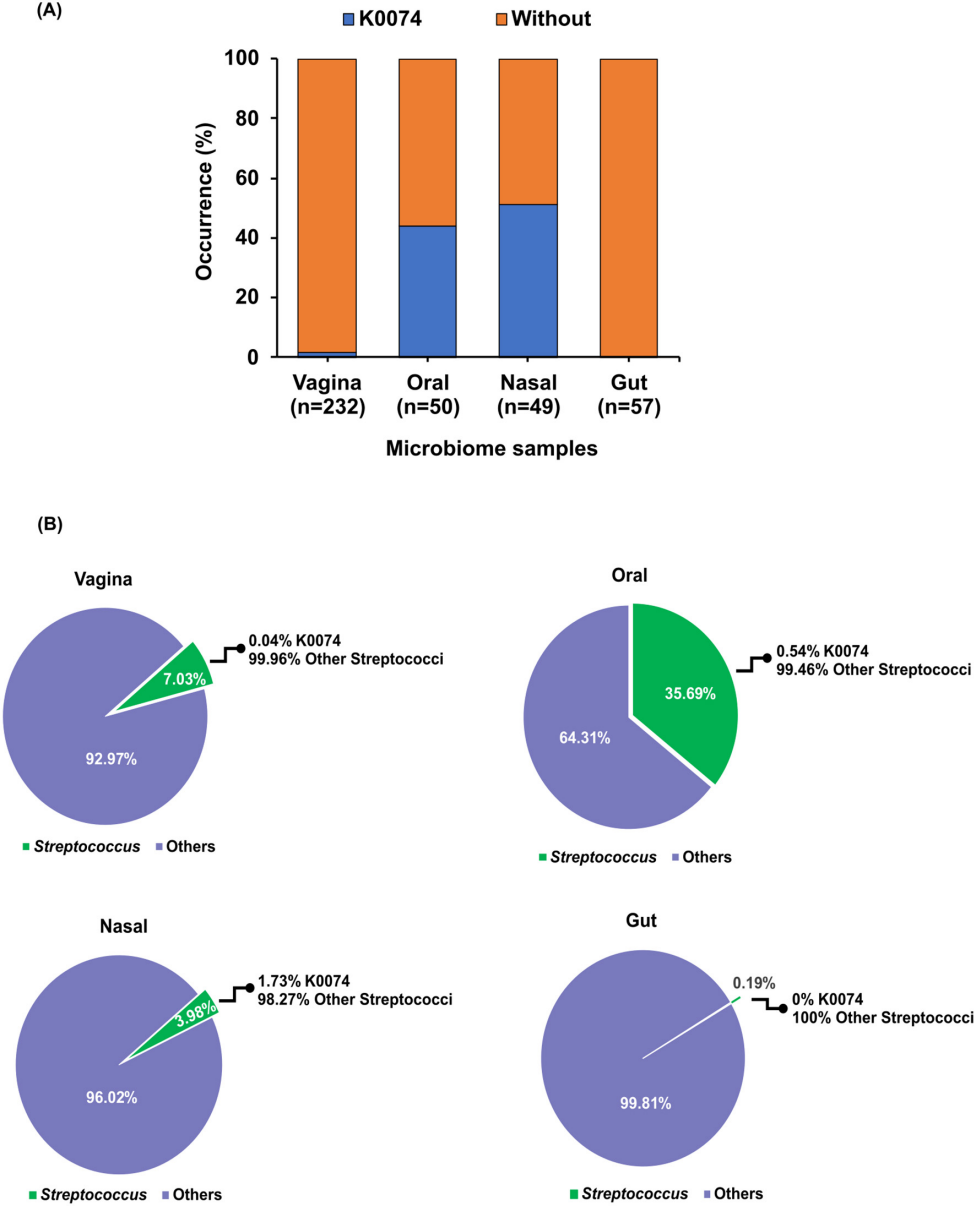
Occurrence of K0074 across human microbiome. **(A)** Occurrence of K0074 detected through 16S rRNA sequences analysis of microbiome samples from different body sites — vagina (n=232), oral (n=50), nasal (*n* = 49), and gut (*n* = 57). **(B)** Abundance of K0074 relative to the other members of the genus *Streptococcus* detected in the microbiome samples from different body sites.

The prevalence and relative abundance of strain K0074 in the human microbiome were assessed through the analysis of 16S rRNA gene sequence datasets from major human body sites—vagina, oral and nasal cavity, and gut. K0074 was commonly detected in the oral and nasal microbiome samples, with occurrence rates of 41 and 51%, respectively (Figure 3A). Despite being first isolated from the vagina, K0074 only demonstrated minimal occurrence rate (1.72%) in the vaginal microbiome samples. Additionally, K0074 was not detected in the gut microbiome samples.

Within the genus *Streptococcus*, K0074 only contributed 0.04%−1.73% to the total abundance of all detected *Streptococcus* species in the analyzed human microbiome samples, excluding those from the gut (Figure 3B). Altogether, these results suggest that strain K0074 is present in various human body sites, representing a minor component of the human microbiome.

### 3.3 K0074 does not induce cytotoxicity and inflammation in human cervical epithelial cells

**Figure 4:**
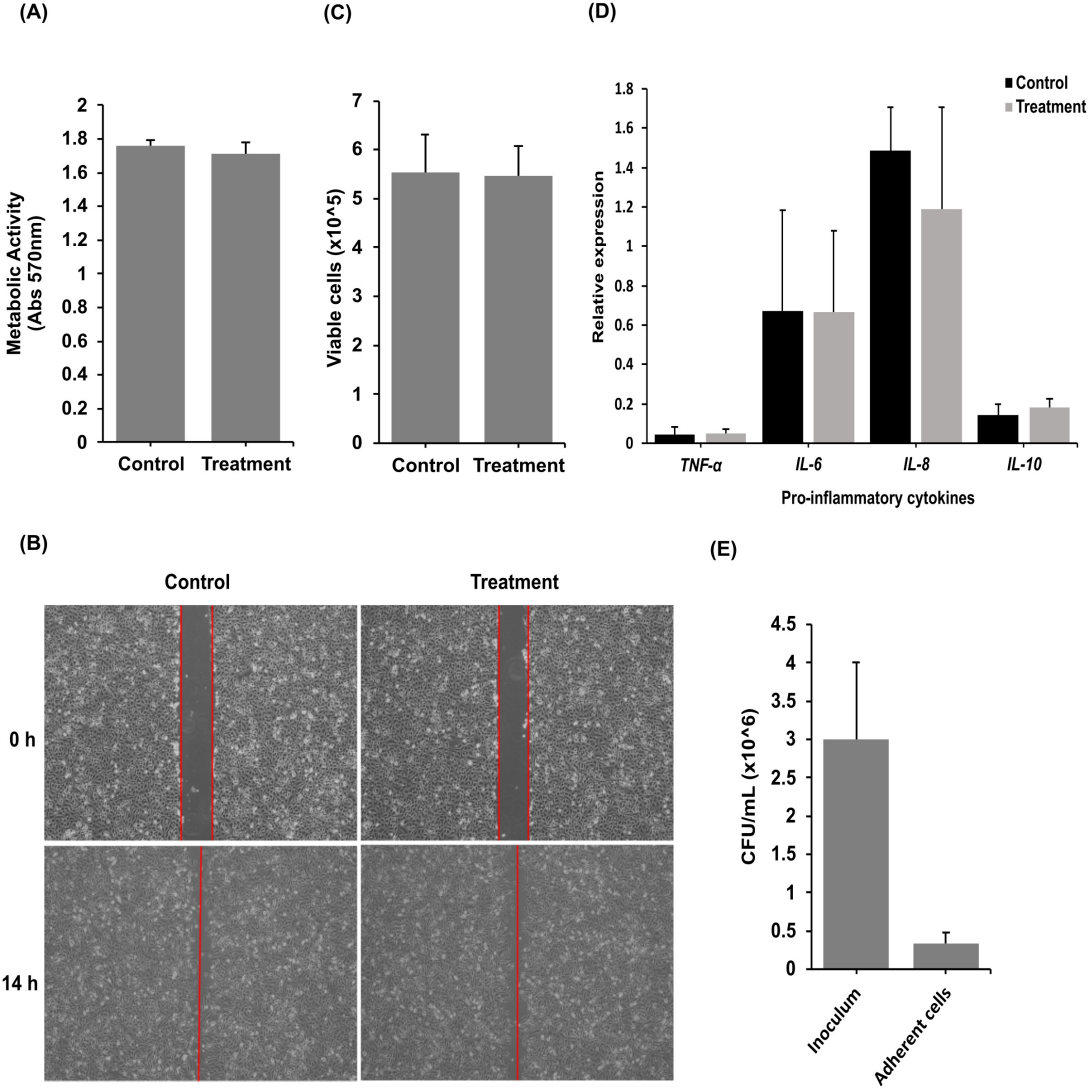
Effects of K0074 on HeLa cells. The CFS of K0074 was used to assess its effect on **(A)** HeLa cell proliferation (cytotoxicity), **(B)** migration, **(C)** membrane integrity (cytolytic), and **(D)** inflammatory response. **(E)** Attachment of K0074 on HeLa cells. Control: medium only.

The members of the genus *Streptococcus* are known to be either pathogenic or commensal bacteria that colonize the human body (Doern and Burnham, 2010; Du Toit et al., 2014; Stoneham et al., 2021). Given that most virulence factors in *Streptococcus* are secreted (Westerlund et al., 2019), we then assessed the effects of K0074 on human cervical epithelial cell line, HeLa cells, using the cell-free supernatant (CFS) of the bacterium. Treatment of HeLa cells using the CFS of K0074 revealed no effects on the viability or proliferation of HeLa cells (Figure 4A), as well as on the cells' migration ability (Figure 4B). The CFS of K0074 also did not lyse or induce cytolytic activity in HeLa cells (Figure 4C). Moreover, no significant induction of inflammatory cytokines gene expressions such as *TNF-*α, *IL-8, IL-6*, and *IL-10* was observed in the CFS-treated HeLa cells compared to the untreated control (medium only) (Figure 4D). In terms of colonization, K0074 demonstrated an adherence efficiency of 11.1% to HeLa cells *in vitro* (Figure 4E). Overall, these results imply that although K0074 exhibits a substantial ability to colonize vaginal epithelial cells, it does not produce secreted cytolytic factors, toxins, or enzymes that affect host cell physiology.

### 3.4 Comparative genomics of K0074 reveals its low pathogenic potential

**Figure 5:**
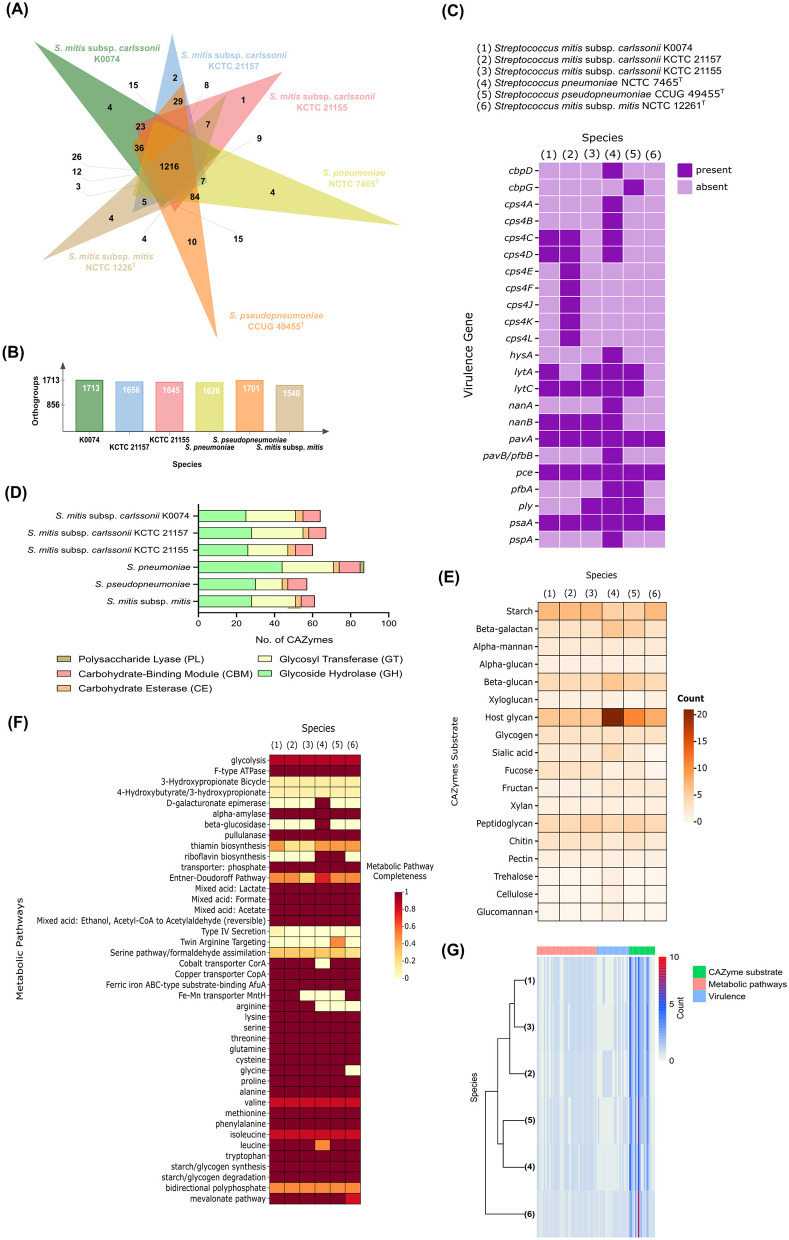
Comparative genomics of K0074 and closely related strains and species. **(A)** Venn diagram showing the orthogroups shared among strains and species. **(B)** Total number of orthogroups detected in each *Streptococcus*. **(C)** Virulence genes identified from the genomes of each *Streptococcus*. **(D)** Identified CAZymes and **(E)** their predicted substrates. **(F)** Metabolic pathways identified from the genomes of each *Streptococcus*. **(G)** Hierarchical clustering of strains and species based on identified metabolic pathways, virulence factors, and CAZyme substrates.

Comparative genomics was performed to further understand the biology of K0074. We compared the genome of K0074 with the genomes of closely related subspecies strains and type species, including two strains of *S. mitis* subsp. *carlssonii* (KCTC 21155 and KCTC 21157), and *S. mitis* subsp. *mitis*, as well as with species known for their pathogenic potentials, such as *S. pneumoniae* and *S. pseudopneumoniae*. Analysis of the orthologous gene clusters (orthogroups) demonstrated high numbers of core orthogroups (1,216) shared by the six species (Figure 5A). The identified orthogroups from each species ranged from 1,540 to 1,713, with K0074 and *S. mitis* subsp. *mitis* having the highest and least numbers of orthogroups, respectively (Figure 5B). The core orthogroups were enriched with genes coding for unknown functions, and genes involved in translation, amino acid metabolism and transport, transcription, nucleotide metabolism and transport, as well as genes governing inorganic ion transport and metabolism (Supplementary Figure 3). Among the six species, *S. pseudopneumoniae* exhibited the highest number of unique orthogroups (10), while K0074 on the other hand, exhibited four unique orthogroups (Figure 5A) which were mostly coding for hypothetical proteins (Supplementary Table 5). Focusing on K0074 (Figure 5A), the species shared the highest dispensable orthogroups with *S. pseudopneumoniae* (36), followed by *S. pneumoniae* (26). The species shared 23, 15, and 12 dispensable orthogroups with *S. mitis* subsp. *carlssonii* KCTC 21155, KCTC 21157, and *S. mitis* subsp. *mitis*, respectively. The orthogroups shared by K0074 with the pathogenic species *S. pseudopneumoniae* and *S. pneumoniae* mainly represented genes that are coding for hypothetical proteins and not for known virulence factors. On the other hand, *S. pneumoniae* and *S. pseudopneumoniae* shared 84 orthogroups (Figure 5A), some of the genes in these orthogroups code for several virulence factors.

Known *Streptococcus* virulence factors were identified in the genome of K0074 (Figure 5C). A total of eight virulence factors were found, which included genes involved in the biosynthesis of capsular polysaccharide (*csp4C* and *csp4D*), expression of cell wall-degrading enzymes 14-beta-N-acetylmuramidase (*lytC*) and autolysin (*lytA*), production of neuraminidase B (*nanB*), and adhesion factors such as fibronectin-binding protein-like protein A (*pavA*), choline binding protein E (*pce*), and manganese-binding adhesion protein (*psaA*). Although the biosynthesis genes for capsular polysaccharide *cps4C* and *cps4D* were identified, the other genes comprising the entire capsular biosynthesis cluster were not found, suggesting an incomplete cluster for capsular biosynthesis. Additionally, *cps4C* and *cps4D* do not encode for the structural components of the bacterial capsule during biosynthesis. Instead, these genes are exclusively involved in the regulation of the biosynthesis process, functioning as regulatory elements (Whittall et al., 2015). On the other hand, no genes responsible for the production of pore-forming toxins, like pneumolysin (*ply*), were identified in the genome of K0074. Among the six analyzed streptococci, only the genomes of *S. mitis* subsp. *carlssonii* KCTC 21155, *S. pneumoniae*, and *S. pseudopneumoniae* were found to harbor the *ply* gene. As expected, the highly pathogenic *S. pneumoniae* exhibited the highest number of identified virulence genes (17) in its genome, encompassing genes involved in enzyme and toxin production, capsule biosynthesis, and adhesion. Compared to other analyzed species, including K0074, *S. pneumoniae* has a higher number of genes associated with toxin production and host colonization or adhesion. *S. mitis* subsp. *mitis*, on the other hand, was found to contain virulence determinants related only to adhesion and colonization (*pavA, pce*, and *psaA*).

Carbohydrate active enzymes (CAZymes) constitute a diverse group of enzymes that catalyze the biosynthesis, modification, and degradation of carbohydrates (Cantarel et al., 2009; Zheng et al., 2023b). We assessed the presence of CAZymes in the genome of K0074 and conducted comparative analyses with the five other species. The different species generally exhibited similar classes of CAZymes but with huge variations in the abundance of genes representing each class (Figure 5D). A total of 64 CAZymes were identified in K0074, with GT (26) and GH (25) as the most represented CAZyme classes. This number of identified CAZymes was comparable to the number of CAZymes identified in the commensal species *S. mitis* subsp. *mitis* (61), and closely related strains of *S. mitis* subsp. *carlssonii* KCTC 21157 (67) and KCTC 21155 (60). Among the analyzed species, the pathogenic *S. pneumoniae* exhibited the highest number of CAZymes (87) with 44 identified genes predicted to encode GH class of enzymes. Additionally, *S. pneumoniae* was the only species to possess PL (2) class of CAZyme. Substrate analysis of the identified CAZymes from all six species revealed a diverse range of substrate specificities (Figure 5E). Each species exhibited varying numbers of CAZymes acting on specific substate. Predictably, *S. pneumoniae* demonstrated a high number of CAZymes that can act on host glycans (21) supporting its pathogenic nature. *S. pseudopneumoniae*, although exhibited the least repertoire of CAZymes (57), emerged to have the second highest number of host glycan-acting CAZymes. The other species and strains, including K0074, exhibited fewer host glycan-acting CAZymes and shared relatively similar CAZyme substrate profiles. All analyzed species possessed CAZymes capable of acting on glycans, glycogen, sialic acid, and fucose substrates—carbon sources commonly utilized by bacteria across various niches of the human body. Metabolic pathway analysis displayed comparatively alike metabolic pathways shared among the six streptococci (Figure 5F), except with some additional identified pathways and enzymes in *S. pneumoniae* and *S. pseudopneumoniae*. All strains possessed complete pathways for acid production (lactate, formate, and acetate), degradation of starch and glycogen, and synthesis of various amino acids. No metabolic pathways unique to K0074 were identified. Hierarchical clustering based on the identified virulence genes, metabolic pathways, and CAZyme substrates revealed the grouping of K0074 alongside closely related strains of *S. mitis* subsp. *carlssonii*, KCTC 21157 and KCTC 21155, reflecting potential functional similarity (Figure 5G). In contrast, *S. pneumoniae* was distantly separated, exhibiting distinct feature profiles from the other species.

**Figure 6:**
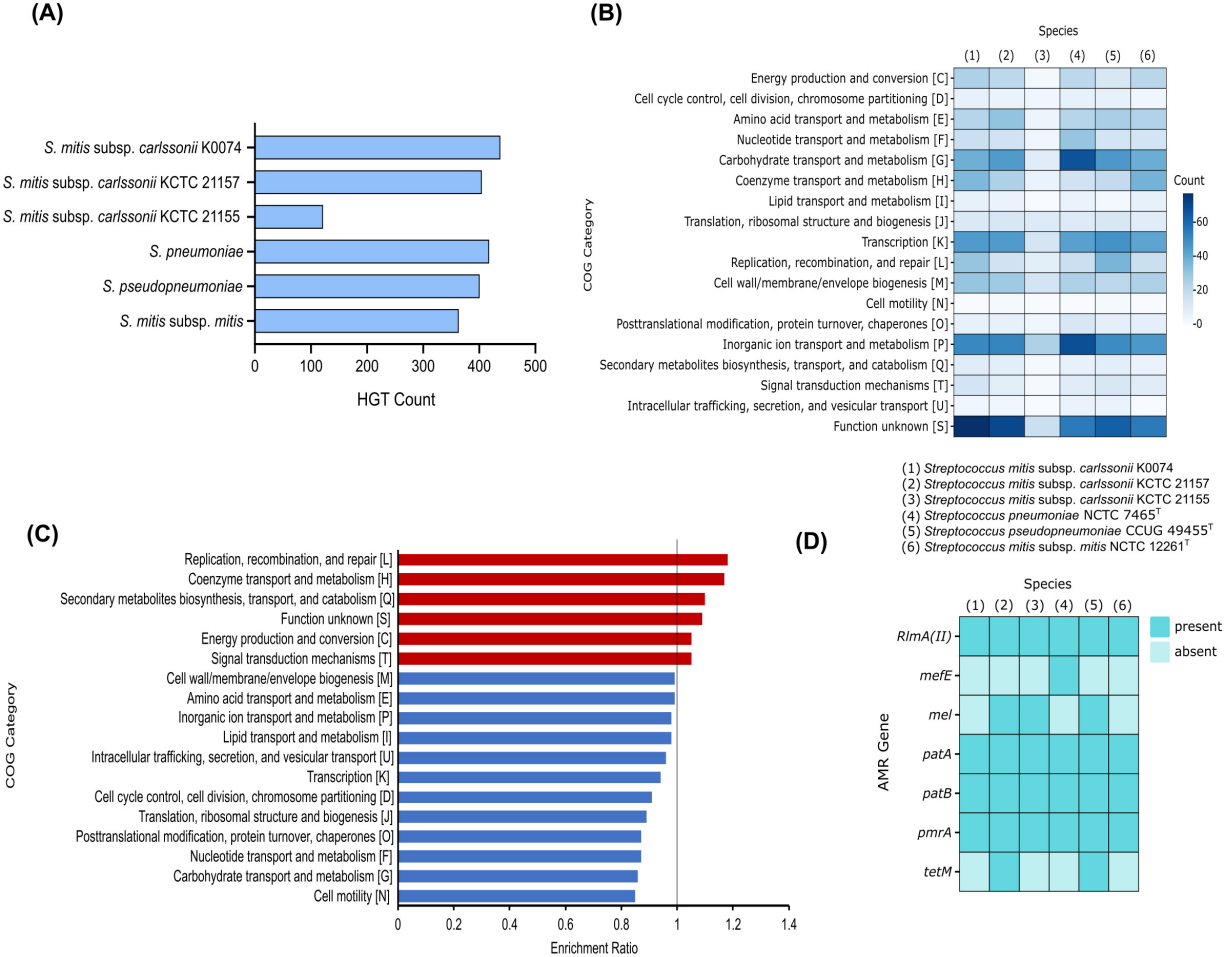
Comparative genomics of K0074 and closely related strains and species. **(A)** Putative HGT genes identified in the genomes of each strain and species. **(B)** COG classification of the identified putative HGT genes. **(C)** Enriched COG categories of K0074 compared to other strains and species. Enrichment ratio > 1 means overrepresented in K0074. **(D)** Identified AMR genes in the genomes of each *Streptococcus*.

Horizontal gene transfer (HGT) is the process by which genetic material is exchanged between organisms. This process is considered a crucial element in the evolution of prokaryotes, playing a significant role in their diversification and adaptation (Wang et al., 2024). We assessed the genome of K0074 for the presence of genes putatively acquired through HGT. As depicted in Figure 6A, K0074 had the highest number of putative HGT genes (437) among all strains analyzed. The putative HGT genes identified in all strains ranged from 121 to 437, with *S. mitis* subsp. *carlssonii* KCTC 21155 having the least. *S. pneumoniae* had the second highest number of putative HGT genes (417). Functional category analysis revealed a huge fraction of putative HGT genes in K0074 (77) belonging to function unknown COG category (Figure 6B). Notably, the putative HGT genes of *S. pneumoniae* were enriched with genes related to inorganic ion transport and metabolism (68) and carbohydrate transport and metabolism COG categories, reflecting the possibly acquired metabolic versatility of the pathogen (Claverys et al., 2000). In comparison to the other species and strains, K0074 exhibited a significantly enriched COG category for replication, recombination, and repair (Figure 6C). This enrichment supports the high number of detected putative HGT genes in its genome, and may indicate a robust capability for genetic diversity.

The genomes of the six streptococci were also analyzed for the presence of antimicrobial resistance (AMR) genes. Multiple AMR genes were identified in each species, ranging from a minimum of four to a maximum of six per species (Figure 6D). K0074 had four identified AMR genes, the *RlmA(II)* coding for a methyltransferase that confers resistance to lincosamide and macrolides, the ABC transporter genes *patA* and *patB* involved in fluoroquinolone resistance, and the *pmrA* gene which encodes a MFS-type efflux pump conferring low level resistance to fluoroquinolones. In addition to these AMR genes, other streptococci including KCTC 21157, KCTC 21155, and *S. pseudopneumoniae* possessed *mel* gene which encodes a homolog of an ABC-F subfamily protein (MsrA) associated with resistance to macrolide, lincosamide, oxazolidinone, phenicol, pleuromutilin, streptogramin, and tetracycline. The AMR gene *mefE*, coding for a proton motive efflux pump involved in macrolide resistance, was only detected in the genome of *S. pneumoniae*. Both KCTC 21157 and *S. pseudopneumoniae* harbored the *tetM* gene, responsible for the synthesis of a ribosomal protection protein that confers resistance to tetracycline. *S. mitis* subsp. *mitis* and K0074 shared similar AMR gene markers, which confer resistance to macrolides, lincosamides, and fluoroquinolone. Antimicrobial susceptibility testing of K0074 (Supplementary Table 6), however, demonstrated sensitivity to macrolides, lincosamides, fluoroquinolones, and most of the common antimicrobials.

Collectively, these results suggest that *S. mitis* subsp. *carlssonii* K0074 may be less pathogenic and metabolically versatile than some other strains and species, such as *S. pneumoniae* and *S. pseudopneumoniae*. However, the new strain still possesses significant genetic potential and adaptability due to its high number of putative HGT genes.

## 4 Discussion

Strain-level characterization has emerged as a critical facet of microbiome research, given that genetically distinct variants within the same species can display markedly different virulence profiles, metabolic potential, and ecological interactions. These intra-species differences profoundly influence host health, therapeutic efficacy, and the stability of microbial communities (Anderson and Bisanz, 2023; Wolff et al., 2023). In this study, we described a new strain of *S. mitis* subsp. *carlssonii*, strain K0074, isolated from the human vagina. The identification of the strain is in accordance with the proposed classification criteria for the *S. mitis* complex recently devised by Kilian et al. (2025). The new classification scheme does not follow the universally accepted thresholds for species delineation—≥70% dDDH and ≥95% ANI (Nouioui and Sangal, 2022; Kilian et al., 2025). This taxonomic shift led to establishment of two novel subspecies *S*. *mitis* subsp. *mitis* and *S. mitis* subsp. *carlssonii* and the reclassification of several established species, including *S. humanilactis* and *S. hohhotensis*.

Strain K0074 has a genome size of 2.08 Mb, which falls within the range of genome sizes (1.51–2.46 Mb) found among known species of *Streptococcus* (Patel and Gupta, 2018). Based on the WGS of the strain, K0074 has sets of enzymes and transporter systems involved in the uptake, biosynthesis, and utilization of carbohydrates, amino acids, and other compounds. Transporters such as the PTS are essential in the metabolic processes of streptococci, which are known to be fermentative bacteria relying primarily on sugars as their main energy source for growth (Leonard and Lalk, 2018; Zuber and Kreth, 2023). Similar with the other streptococci, common major pathways for carbon metabolism, such as the glycolytic EMP pathway and pentose phosphate pathway are present in K0074. The production of lactate and other acids from carbohydrate metabolism is also inscribed in the genome of the strain, reflecting the inherent ability of LAB (Du Toit et al., 2014; Wang et al., 2021). Another shared metabolic aspect of streptococci found in K0074 is the lack of complete TCA cycle (Leonard and Lalk, 2018). Streptococci lack a complete TCA cycle due to their primary reliance on carbohydrate fermentation for energy production (Zeng et al., 2023). Although the absence of a TCA cycle may appear to significantly impact amino acid biosynthesis, given that the intermediates of the pathway are essential for the *de novo* synthesis of many amino acids (Leonard and Lalk, 2018), K0074 is still predicted to synthesize a range of amino acids through alternative metabolic pathways. On the other hand, the strain is also endowed with pullulanase, an enzyme responsible for the breakdown of glycogen (Naik et al., 2023). Glycogen is the major carbon source for microorganisms in the vagina, produced primarily by the vaginal epithelial cells (Mirmonsef et al., 2016). Given the isolation ecology of the strain, the presence of a glycogen-degrading enzyme may significantly enhance its survival in the highly dynamic vaginal microenvironment.

VGS are typically found in various key regions of the human body, such as the oral cavity, respiratory, gastrointestinal, and urogenital tracts (Doern and Burnham, 2010; Du Toit et al., 2014). Although K0074 was initially isolated from the vagina, our results indicate that this strain is also present in other parts of the human body and is more prevalent in the oral and nasal microbiome. This finding is not surprising, as members of the VGS are predominantly found inhabiting the oral cavity, where they play significant roles in maintain homeostasis and overall oral health (Baty et al., 2022; Bloch et al., 2024). Streptococci are also common members of the nasal microbiome, along with staphylococci and corynebacteria (Thangaleela et al., 2022). The microbiome compositions of the oral and nasal cavity have been shown to have greater similarity, likely due to direct microbial exchange (Zhou et al., 2024) which probably explains the high occurrence of K0074 in these body sites. In contrast to their abundance in the oral cavity, VGS constitute a minor population in the vaginal microbiome, in which *Lactobacillus* species dominate (Anahtar et al., 2018). The role of K0074, and the VGS in general, in the vaginal microbiome remains largely underexplored. However, previous study of our strain K0074 demonstrated protective effects against the colonization of *Staphylococcus aureus* in the vaginal epithelium, underscoring the possible accessory role of the strain in modulating the vaginal microbiome (Montecillo et al., 2024).

Several members of the genus *Streptococcus* are known to cause a wide variety of infections in human (Leonard and Lalk, 2018; Brokaw et al., 2021; Lannes-Costa et al., 2021; Stoneham et al., 2021; Nanayakkara et al., 2023). These pathogenic members harbor an arsenal of virulence factors that aid in their colonization, invasion, evasion of host immune responses, and contribute to their overall pathogenesis (Westerlund et al., 2019; Pattnaik et al., 2020; Lannes-Costa et al., 2021). Our *in vitro* virulence characterization and comparative genome-level analyses of K0074 revealed valuable insights into several aspects of the strain's biology. Unlike the known closely related pathogen *S. pneumoniae*, K0074 lacks secreted cytolytic proteins and toxins, rendering no negative effects on human cells *in vitro*. This observed phenotype is further substantiated by the absence of the *ply* gene in the genome of K0074. The *ply* gene encodes the secreted pore-forming toxin pneumolysin in *S. pneumoniae* (Nishimoto et al., 2020), which is also present in other pathogenic members of the mitis subgroup, such as *S. pseudopneumoniae* (Morales et al., 2015). The absence of the *ply* gene in K0074 suggests a reduced potential for cytotoxicity, consistent with its observed lack of deleterious effects on human cells *in vitro*. Moreover, K0074 has fewer virulence factors in its genome compared to *S. pneumoniae*. Several of which are adhesion factors, which might have facilitated the observed *in vitro* adhesion or colonization of the strain on the host epithelial surface.

In contrast to *S. pneumoniae*, K0074 harbors a smaller repertoire of CAZymes, particularly those that target host-derived carbohydrate sources like glycans. In microorganisms CAZymes play crucial roles in many biological processes, including metabolism, colonization, and infection by facilitating the biosynthesis, modification, and degradation of carbohydrates (Velez-Cortes and Wang, 2022; Crouch, 2023; Pena et al., 2024). Given the lack of TCA cycle in streptococci, they strictly rely on carbohydrates for energy production (Leonard and Lalk, 2018). However, readily available carbohydrates are limited in several niches of the human host that most streptococci colonize. In these environments, some streptococci rely on host-glycan or possibly on other microbes for metabolism (Andreassen et al., 2020). Glycans, which can be simple or complex carbohydrates, are ubiquitous in many biological systems and are predominantly found decorating the surfaces of cells (Varki, 2017; Lundstrøm and Bojar, 2022). Glycans in the form of mucins in mucosal surfaces in some areas of the human body act as protective barriers, physically inhibiting the invasion of pathogenic microorganisms to the underlying membrane (McGuckin et al., 2011). The degradation and subsequent utilization of surface host glycans as nutrients can be viewed as a strategy employed by many pathogens to gain access to host cells, thereby facilitating invasion and progression into a disease state (Marcobal et al., 2013; Agarwal et al., 2023). The extensive repertoires of GHs and host glycan-degrading CAZymes of *S. pneumoniae* clearly indicate the pathogenic nature of the species. Indeed, various studies have associated the ability of *S. pneumoniae* to degrade glycans as an important virulence trait, facilitating invasion and survival (Terra et al., 2015; Andreassen et al., 2020). On the other hand, the limited collection of CAZymes targeting host glycan substrates in K0074 suggests a restricted ability to exploit host carbohydrates relative to *S. pneumoniae*. This limited ability to degrade and utilize host glycans may be an indicative of a reduced ability to establish and maintain infection, reflecting the low virulence and restricted pathogenic potentials of K0074.

Members of the mitis phylogenetic cluster are known to undergo genetic competence/natural transformation, a state/process by which bacteria can actively uptake and incorporate naked extracellular DNA from the environment (Salvadori et al., 2019). Natural transformation is one of the key drivers of evolution, promoting genetic variability through HGT. In streptococci, HGT plays important part in their genetic variability, particularly in relation to virulence (Salvadori et al., 2019). Indeed, here we showed several genes putatively acquired through HGT in the genomes of streptococci, including K0074. These genes might have been acquired through natural transformation, or other routes of HGT. Notably, K0074 has the highest number of putative HGT genes, which suggest potential genetic diversity. However, majority of these HGT genes have unknown function, and their acquisition might not have a significant impact on the virulence and survival of the strain. While HGT can significantly alter bacterial genomes, in fact, not all transfer events are biologically significant. Some may simply be part of a continual evolutionary process that only occasionally yields beneficial outcomes (Arnold et al., 2021). Conversely, in the pathogenic *S. pneumoniae*, the putative HGT genes represent genes involved in transport and metabolism, reflecting the possible beneficial outcomes of the HGT events and the acquired metabolic versatility of the pathogen. Interestingly, the streptococci analyzed in this study share 71–79% of orthologous genes which highlights their high genetic similarities. This similarity might have been orchestrated by extensive recombination events within the mitis cluster during parallel evolution which have been previously reported (Salvadori et al., 2019). Although these streptococci share high genetic similarity, differences in their pathogenic potentials are still strikingly evident.

A number of AMR genes were identified in the genome of K0074 as well as in the genomes of the other members of the mitis group analyzed in this work. Majority of their AMR genes are associated with resistance to macrolides, lincosamides, and fluoroquinolones, highlighting the prevalence and diversity of mechanisms that target these specific antibiotic classes within streptococci. The presence of these AMR genes is not surprising, considering that these classes of antibiotics are routinely employed in the treatment of infections caused by streptococci (Haenni et al., 2018; Berbel et al., 2022). Members of the VGS are also frequently identified to have macrolide and fluoroquinolone resistance determinants (Alves-Barroco et al., 2020; Berbel et al., 2022). The frequent use of these classes of antibiotics likely exerts selective pressure on bacterial populations, promoting the acquisition, retention, and spread of the resistance genes. Transposons or integrative and conjugative elements might have also disseminated the resistance determinants among these streptococci, which have been previously reported (Haenni et al., 2018; Alves-Barroco et al., 2020; Berbel et al., 2022). Although K0074 harbors these AMR genes, the strain remains susceptible to the corresponding antimicrobial agents. This phenomenon, where AMR genes are present but not expressed, has been reported in some bacteria and is referred to as silent or cryptic AMR genes (Deekshit and Srikumar, 2022). In some cases, AMR genes also require the involvement of accessory genes (non-classical AMR genes) to fully exert or present the resistance phenotypes (Dillon et al., 2024). The observed antibiotic response of K0074 might stem from these silent AMR genes or the lack of necessary accessory genes, resulting in the susceptibility of the strain to the corresponding antimicrobial agents despite harboring the resistance determinants. The presence of these AMR genes in K0074, even though they may be silent, still poses a significant concern as the species may serve as a reservoir of antimicrobial resistance genes. These genes can potentially be activated under favorable conditions or disseminated to other bacteria, thereby contributing to the spread of antimicrobial resistance.

The isolation of K0074 from an endometrial cancer patient raises interesting clinical considerations. Recent studies have shown that alterations in the vaginal microbiome composition are associated with various gynecological cancers, including endometrial cancer (Łaniewski et al., 2020; Javadi et al., 2024). While K0074 is primarily detected in oral and nasal microbiomes, its presence in the vaginal tract suggests possible niche adaptation. Further studies with larger cohorts of patients would be valuable to understand the distribution and potential roles of this strain across in both healthy individuals and those with reproductive tract conditions.

While the present study provides phenotypic and genomic profiles of *S. mitis* subsp. *carlssonii* K0074, reliance on this single strain—compounded by its documented occurrence in other body sites—limits the strength of our findings. Broader strain isolation and strain-level comparative characterization will be essential to expand these findings and to uncover broader ecological or clinical relevance.

## 5 Conclusion

Based on our analyses, we conclude that K0074 represents a distinct strain of *S. mitis* subsp. *carlssonii*. The strain exhibits low virulence and pathogenic potentials and can therefore be considered a commensal member of the human microflora. However, the high number of HGT genes in the genome of the strain may indicate significant genetic potential and adaptability, warranting further investigation into their specific roles and implications.

## Data Availability

The datasets presented in this study can be found in online repositories. The names of the repository/repositories and accession number(s) can be found in the article/Supplementary material.
