# Supplementary material for: Characterization of Streptococcus mitis subsp. carlssonii isolated from human vagina: prevalence, phenotypic, and genomic insights
Source: Front Microbiol. 2025 Aug 25;16:1625724. doi: 10.3389/fmicb.2025.1625724 (PMC12415561; doi:10.3389/fmicb.2025.1625724)
Supplement: Supplementary file 1 [file Supplementary_file_1.docx]

Supplementary Material

# Supplementary Figures and Tables

## Supplementary Figures


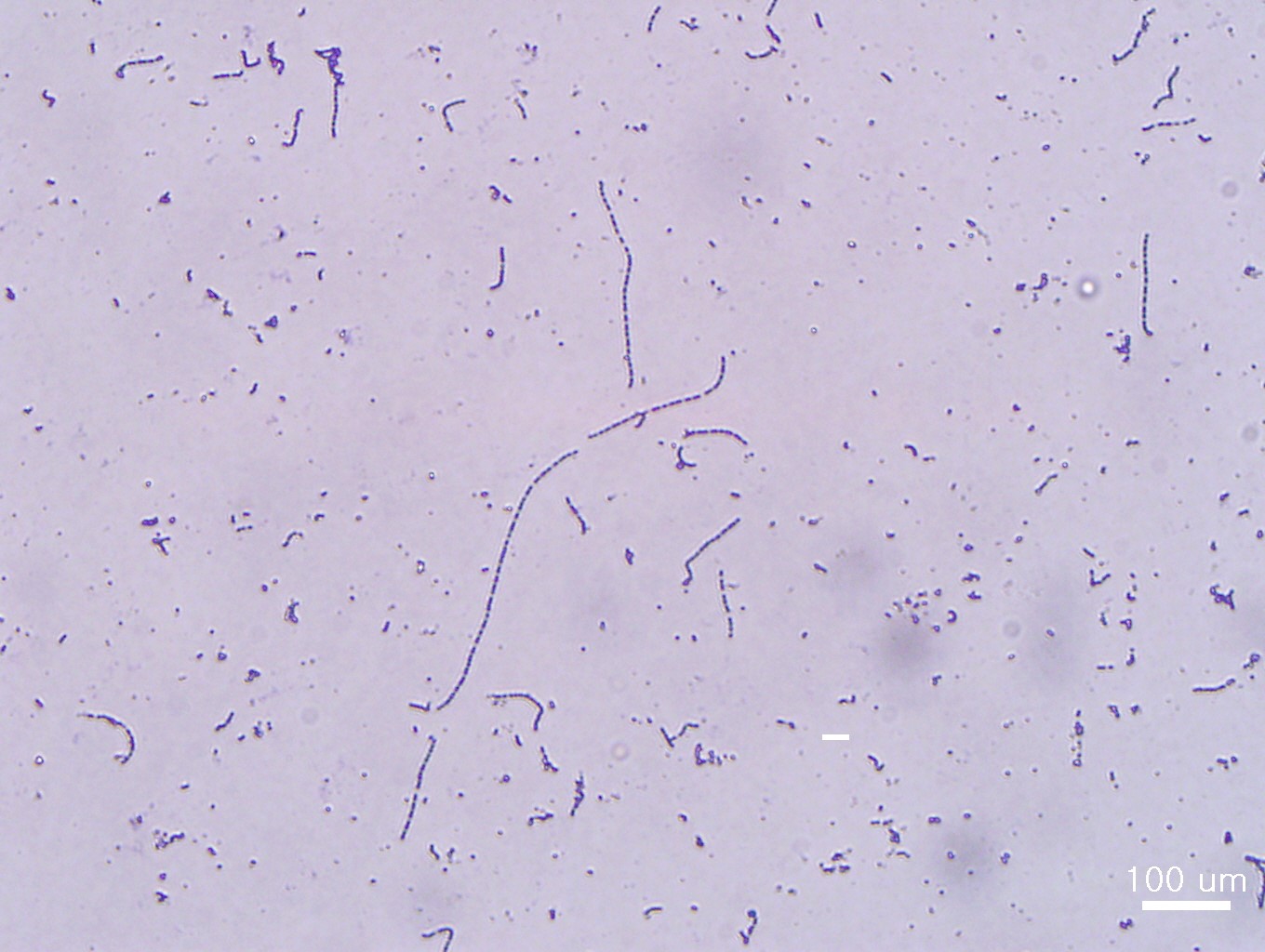


**Supplementary Figure 1.** Gram stain reaction of K0074. Scale bar 30µm.


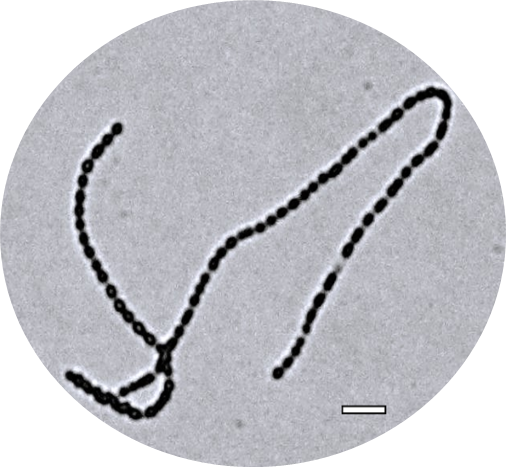


**Supplementary Figure 2.** Cell morphology and arrangement of K0074. Scale bar 5µm.


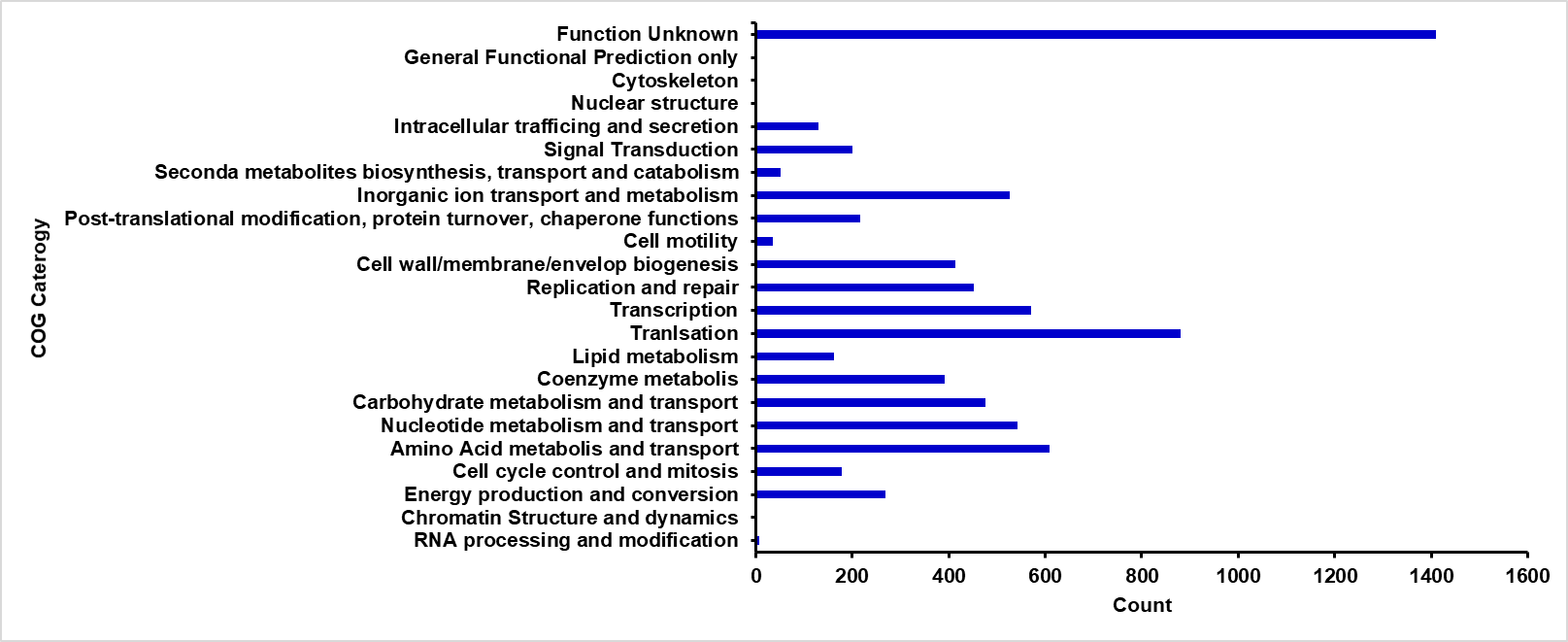


**Supplementary Figure 3.** COG assignements of the core orthogroups shared by all streptococci included in the analysis.

## Supplementary Tables

**Supplementary Table 1.** List of oligonucleotides used in the qRT-PCR analysis.

| Gene | Gene accession no. | Forward | Reverse |
| --- | --- | --- | --- |
| TNF-α | NM_000594 | ATCAGAGGGCCTGTACCTCA | GGAAGACCCCTCCCAGATAG |
| IL-8 | NM_000584 | AGGGTTGCCAGATGCAATAC | CCTTGGCCTCAATTTTGCTA |
| IL-6 | NM_000600 | TACCCCCAGGAGAAGATTCC | TTTTCTGCCAGTGCCTCTTT |
| IL-10 | NM_000572 | AGGGCACCCAGTCTGAGAAC | TGGCAACCCAGGTAACCCTT |
| GAPDH | NM_002046 | ACAGTCAGCCGCATCTTCTTTTGC | TTGAGGTCAATGAAGGGGTC |

**Supplementary Table 2.** Microbiome Datasets (oral, nasal, gut) SRA Accession Numbers.

| Oral n=50 | Nasal n=49 | Gut n=57 |
| --- | --- | --- |
| SRR31517015 | SRR26333803 | SRR31639103 |
| SRR31517016 | SRR26333802 | SRR31639114 |
| SRR31517017 | SRR26333804 | SRR31639125 |
| SRR31517018 | SRR26333807 | SRR31639161 |
| SRR31517019 | SRR26333809 | SRR31639162 |
| SRR31517020 | SRR26333810 | SRR31639048 |
| SRR31517021 | SRR26333814 | SRR31639059 |
| SRR31517022 | SRR26333811 | SRR31639070 |
| SRR31517023 | SRR26333813 | SRR31639081 |
| SRR31517024 | SRR26333812 | SRR31639092 |
| SRR31517025 | SRR26333815 | SRR22330030 |
| SRR31517026 | SRR26333816 | SRR22330058 |
| SRR31517027 | SRR26333921 | SRR22330056 |
| SRR31517028 | SRR26333821 | SRR22330057 |
| SRR31517029 | SRR26333826 | SRR22330054 |
| SRR31517030 | SRR26333820 | SRR22330055 |
| SRR31517031 | SRR26333829 | SRR22330052 |
| SRR31517032 | SRR26333823 | SRR22330053 |
| SRR31517033 | SRR26333825 | SRR22330043 |
| SRR31517034 | SRR26333824 | SRR22330044 |
| SRR31517035 | SRR26333839 | SRR22330041 |
| SRR31517036 | SRR26333832 | SRR22330042 |
| SRR31517037 | SRR26333831 | SRR22330039 |
| SRR31517038 | SRR26333833 | SRR22330040 |
| SRR31517039 | SRR26333805 | SRR22330038 |
| SRR31517040 | SRR26333837 | SRR22330051 |
| SRR31517041 | SRR26333836 | SRR22330049 |
| SRR31517042 | SRR26333923 | SRR22330050 |
| SRR31517043 | SRR26333846 | SRR22330047 |
| SRR31517044 | SRR26333848 | SRR22330048 |
| SRR31517045 | SRR26333844 | SRR22330045 |
| SRR31517046 | SRR26333834 | SRR22330046 |
| SRR31517047 | SRR26333845 | SRR22330028 |
| SRR31517048 | SRR26333850 | SRR22330029 |
| SRR31517049 | SRR26333822 | SRR22330024 |
| SRR31517050 | SRR26333911 | SRR22330025 |
| SRR31517051 | SRR26333908 | SRR22330026 |
| SRR31517052 | SRR26333910 | SRR22330027 |
| SRR31517053 | SRR26333919 | SRR22330020 |
| SRR31517054 | SRR26333909 | SRR22330021 |
| SRR31517055 | SRR26333913 | SRR22330018 |
| SRR31517056 | SRR26333912 | SRR22330019 |
| SRR31517057 | SRR26333916 | SRR22330016 |
| SRR31517058 | SRR26333851 | SRR22330017 |
| SRR31517059 | SRR26333918 | SRR22330014 |
| SRR31517060 | SRR26333828 | SRR22330015 |
| SRR31517061 | SRR26333920 | SRR22330012 |
| SRR31517062 | SRR26333914 | SRR22330013 |
| SRR31517063 | SRR26333907 | SRR22330010 |
| SRR31517064 |  | SRR22330011 |
|  |  | SRR22330009 |
|  |  | SRR22330008 |
|  |  | SRR22330007 |
|  |  | SRR22330006 |
|  |  | SRR22330005 |
|  |  | SRR22330004 |
|  |  | SRR22330003 |

**Supplementary Table 3.** Genome sequences used in the study.

| Species | Strain | Type | Accession no. |
| --- | --- | --- | --- |
| *Streptococcus humanilactis* | IMAU99125 | yes | GCF_019448275.1 |
| *Streptococcus pseudopneumoniae* | CCUG 49455 | yes | GCF_002087075.1 |
| *Streptococcus gwangjuense* | KCOM 1679 | yes | GCF_003627155.1 |
| *Streptococcus vulneris* | DM3B3 | yes | GCF_019218685.1 |
| *Streptococcus toyakuensis* | TP1632 | yes | GCF_024346585.1 |
| *Streptococcus hohhotensis* | IMAU 99199 | yes | GCF_020089495.2 |
| *Streptococcus mitis* | NCTC 12261 | yes | GCF_000148585.2 |
| *Streptococcus pneumoniae* | NCTC7465 | yes | GCF_001457635.1 |
| *Streptococcus chosunense* | ChDC B353 | yes | GCF_003626515.1 |
| *Streptococcus oralis* | NCTC11427 | yes | GCF_900637025.1 |
| *Streptococcus downii* | CECT 9732 | yes | GCF_004353325.1 |
| *Streptococcus infantis* | ATCC 700779 | yes | GCF_000187465.1 |
| *Streptococcus rubneri* | DSM 26920 | yes | GCF_004785935.1 |
| *Streptococcus australis* | ATCC 700641 | yes | GCF_000186465.1 |
| *Streptococcus sanguinis* | ATCC 10556 | yes | GCF_000194945.1 |
| *Streptococcus peroris* | ATCC 700780 | yes | GCF_000187585.1 |
| *Streptococcus ilei* | I-G2 | yes | GCF_000479335.1 |
| *Streptococcus lactarius* | CCUG 66490 | yes | GCF_016642265.1 |
| *Streptococcus cristatus* | ATCC 51100 | yes | GCF_000222765.1 |
| *Streptococcus parasanguinis* | ATCC 15912 | yes | GCF_000164675.2 |
| *Streptococcus gordonii* | CCUG 33482 | yes | GCF_001553855.1 |
| *Streptococcus panodentis* | CCUG 70867 | yes | GCF_017884005.1 |
| *Streptococcus porcorum* | DSM 28302 | yes | GCF_040545265.1 |
| *Streptococcus salivarius* | NCTC 8618 | yes | GCF_000785515.1 |
| *Enterococcus faecalis* | DSM 20478 | yes | GCF_029024925.1 |

**Supplementary Table 4.** 16S rRNA sequence similarity values of strain K0074 shared with closely related strains and species of *Streptococcus*.

| **Species** | **Strain** | **Similarity (%)** | **Isolation Source** |
| --- | --- | --- | --- |
| *Streptococcus* sp*.* | 321A | 99.93 | Human pharynx |
| *Streptococcus mitis* subsp*. carlssonii* | KCTC 21155 | 99.85 | Human breast milk |
| *Streptococcus mitis* subsp*. carlssonii* | KCTC 21157 | 99.78 | Human breast milk |
| *Streptococcus pseudopneumoniae* | ATCC BAA-960^T^ | 99.59 | Human sputum |
| *Streptococcus toyakuensis* | TP1632^T^ | 99.59 | Human blood |
| *Streptococcus mitis* subsp*. mitis* | NCTC 12261^T^ | 99.52 | Human oral cavity |
| *Streptococcus pneumoniae* | NCTC 7465^T^ | 99.38 | unknown |
| *Streptococcus oralis* | ATCC 35037^T^ | 98.91 | Human mouth |
| *Streptococcus infantis* | ATCC 700779^T^ | 98.64 | Human pharynx |
| *Streptococcus rubneri* | LMG 27207^T^ | 98.42 | Human throat |
| *Streptococcus australis* | ATCC 700641^T^ | 98.16 | Human saliva |

**Supplementary Table 5.** Genes/proteins unique to K0074 (in comparison to the other strains/species used in the analyses).

| **Sequence** | **Accession** | **Annotation** |
| --- | --- | --- |
| >K0074 | FCAKDBHC_00393 | hypothetical protein |
| MRKEEKTRLRREKIITAALFEFATKGYQGFVINELCKVDGISKGVLYHNF |  |  |
| SGKSDLYLTCFQESFEKALAVFLGVEGQVPSLADYMERRHQFYQQYPEHS |  |  |
| HIFFEAMIATPEELEADIAPQKAIFLDLNEQVCQKLISESKSYLFGLE |  |  |
| >K0074 | FCAKDBHC_00394 | hypothetical protein |
| MNKFVKNLYQNPKAIFLDLNEQVCQKLISESKLKEHIDEKRAMDYLRLIQ |  |  |
| DMFRSYYLTVSSDSSLPDLVSGYEHQLSQVLDMMIYGILEEDSSKKGEK |  |  |
| >K0074 | FCAKDBHC_00395 | hypothetical protein |
| MSKIWKWLLLIAGIFAVFVGFNMFAHPLISLASMTFWFALVFAVQGISEI |  |  |
| VQYFKSEEKHGWNLFGGIVTLILALTLFSGSFIEMVTFVPFIISLWALTN |  |  |
| GITKTIVGFKVRKTDKSVGTPLVWMGILGIVAGLIMMGHPLMTGLYISYT |  |  |
| IAFVFIYQGIVAIVQFFKIK |  |  |
| > K0074 | FCAKDBHC_01294 | hypothetical protein |
| MSKKLSWLSIVAGVLSILAGFYLMANPALSLLSFAILFALIFMVNGISDI |  |  |
| IKYFSADEKSGWDLFTGVMTVLLAIWLFSGTFFEKVTFIPFIFAFWALFT |  |  |
| GVSKTIMSFEVKKVDKKLGSTLLWTGILGIIAGIIMMGHPLMTGVIVTYT |  |  |
| VAFVFIYQGIAAIVLYFKSKKA |  |  |
| > K0074 | FCAKDBHC_01295 | hypothetical protein |
| MKKELKTQLTRERIIEAALIEFSQKGYKAFGINELCKNHKISKGILYHNF |  |  |
| SGKTELYLACVRESFQKAVSIIRGESGDIPSLADYMERRHRFSKDFPHHS |  |  |
| HVFFEVWMTAPAEIAEEVAQEKAVFEDLNRQVSEKLLSESTLKDYISQEA |  |  |
| ALYYLTFIQQLFRSYYLGPADLKEPAVWPANMKMTSKRSFIS |  |  |
| > K0074 | FCAKDBHC_01339 | hypothetical protein |
| MFIWDWVSILFGWIIFFALISFVIIKLFEVISTLKVGIECKKKLKQLKNK |  |  |
| > K0074 | FCAKDBHC_01343 | hypothetical protein |
| MVKHGKFVSQAAEAEKYYRNENDIKRKRKPADKKGAENEAKAEDNAFRNA |  |  |
| DNRISHNWHQLLLDQKKAYALTYPPTFDVDDKSVNDKIVDVLGDDYERIS |  |  |
| KQLCVNAGNAGIAWLHVWKDASDNSFRYACVDSKEVIPIYSKSLDKKLIG |  |  |
| VLRVYSSIDETDGKNYTVYEYWNDKECSFYRHEKEKPLEELEPFQAISLI |  |  |
| DTMNGDRSSDNSFEHDFGLVPFIPFKNNEIETNDLKPIKDLVDVYDKVFS |  |  |
| GFVNDTDDVQEVIFVLTNYGGQDKQEFLEDLKRYKMIKMDNDGMGDQSGV |  |  |
| TTIAIDIPTEARNLILERTKKQIFISGQGVNPETDKLGNSSGVALKFLYS |  |  |
| LLELKAGNMETQFRSGYATLVKMILRHLGLSDKLKIKQTWTRNSINNDTE |  |  |
| MAQVVSTLATITSRENVAKSNPIVEDWQDELRLQKADQEEQSEKLYDMEE |  |  |
| VEHESETE |  |  |
| > K0074 | FCAKDBHC_01565 | hypothetical protein |
| MFIWEWVSIAFGWLVFLLLIFIILAVISGIIESVKKELKK |  |  |
| > K0074 | FCAKDBHC_01569 | hypothetical protein |
| MYEHLTYPRDGYDEGSLKKDLIYKLITKHSTEGSHLKKLKSYYMGEHAIL |  |  |
| NHKRRNVNAPNYKTVANHAKDIADTATGYFMGNPIKYNNTAEGDIDELLT |  |  |
| AFDGAEIDQVDAQNALNMAIYGRAYEYIYAKEGLTELDSTSIDPENTFMV |  |  |
| YDDSIERKPLFAVYYYQVKDDTKDTTKYQAEVFTENLHYHMVLRSTDAGT |  |  |
| TQNEQVEAHNLGQIPIIEYRNNHFAIGDYEQQISLIDAYNSLMGNRVNDK |  |  |
| EQAVESILVLYGTQLADTPEDAKVAMKILSEEGLLELPGDSARAEFLKNT |  |  |
| LDESATEILRTALKEDIYTFSHVPNLTDENFAGNTSGVAMEFKLMGLEMI |  |  |
| TKTKEANYKRGLRQRIAIFAHYLGMKQIALESHSIVPQFSRGLPKNLLEI |  |  |
| SQIVNNLEGKVTNRQLISLLPFVEDPDAELEALEEEKKKNMEDMPMFNKD |  |  |
| NTKPEDEVEDEESGVLGEEESQSDLPADGQGRKAGRPVR |  |  |

**Supplementary Table 6.** Antibiotic susceptibility profile of K0074.

| Antimicrobial | MIC | Interpretation |
| --- | --- | --- |
| Benzylpenicillin | 0.25 | I |
| Ampicillin | 0.5 | I |
| Cefotaxime | ≤ 0.12 | S |
| Ceftriaxone | 0.25 | S |
| Levofloxacin | 0.5 | S |
| Moxifloxacin | 0.12 | S |
| Erythromycin | ≤ 0.12 | S |
| Clindamycin | ≤ 0.25 | S |
| Linezolid | ≤ 2.0 | S |
| Vancomycin | 0.5 | S |
| Tigecycline | ≤ 0.06 | S |
|  |  |  |
|  |  |  |
| S: Sensitive |  |  |
| I: Intermediate |  |  |
| R: Resistant |  |  |
